# Risk of cancer incidence and mortality in patients with chronic pain: A systematic review and meta-analysis

**DOI:** 10.1007/s10555-026-10341-2

**Published:** 2026-05-11

**Authors:** Chun Hei Lai, Natalia Hefteh, Sophie Kingsford, Adam La Caze, Mahdi Sheikh, Shakti Shrestha, Marie-Odile Parat

**Affiliations:** 1 School of Pharmacy and Pharmaceutical Sciences, The University of Queensland, Dutton Park, Woolloongabba, QLD 4102 Australia; 2 Genomic Epidemiology Branch, International Agency for Research on Cancer (IARC-WHO), Lyon, France

**Keywords:** Cancer mortality, Cancer Incidence, Chronic pain, Fibromyalgia

## Abstract

Evidence from experimental studies suggests that pain may influence cancer development. We conducted a systematic review (registration osf.io/ms437) of the epidemiological studies assessing whether chronic pain, with minimal or non-inflammatory features, increases the risk of cancer incidence or mortality. The search used Scopus and Web of Science up to 11/03/2025. Quality assessment used the Newcastle–Ottawa Scale. Study characteristics and outcomes were analysed using narrative synthesis, with random-effects meta-analysis and meta-regression conducted where appropriate. A total of 23 studies (12 prospective, 11 retrospective) met the inclusion criteria, of which six were rated as high quality. The outcome data synthesis indicates a small increase in cancer mortality rate ratios, and a potential increase in a subset of organs. A total of three body site-specific analyses suggest that chronic pain may be associated with higher incidence for breast (SIR 2.12–4.8) and prostate (SIR 1.49–5.59) cancers, and with increased mortality for lung cancer (MRR 3.09, 95% CI 1.45–6.62). Ten studies were included for meta-analysis, including four studies rated as high quality. Meta-analysis of hazard ratios showed no association between cancer-related mortality and pain (HR 1.00, 95% CI 0.97–1.04), with consistent estimates across pain subtypes in meta-regression. Our review and meta-analysis do not support an overall association between chronic pain and increased risk of cancer mortality or incidence and highlight multiple methodological and conceptual obstacles to exploring this relationship, including reverse causation and confounding factors such as pain and opioid use association. High-quality studies that rigorously address reverse causation and confounding are warranted.

**Supplementary Information:**

The online version contains supplementary material available at 10.1007/s10555-026-10341-2.

## Introduction

The possibility that pain may promote the occurrence of cancer is a growing concern grounded in multiple lines of evidence. Preclinical studies have demonstrated that subjecting rodents to both pain and tumour inoculation increased tumour growth and metastasis. For example, in a study, localised tumour growth in mouse paws caused pain, and pharmacological pain relief or neurectomy decreased metastasis [1]. Similarly, experimental metastasis markedly increased in rats subjected to pain-producing surgical stress, and this was prevented by administration of analgesic opioids, implying a role for pain [1, 2]. In another study, tumour growth was dramatically increased in several murine models of experimental pain, namely neuropathic pain, persistent postsurgical pain, and activation of sensory neurons [3]. These experimental models are designed to demonstrate an effect of acute pain on existing cancer. Whether long term exposure to chronic pain elicits the occurrence of cancer is challenging to demonstrate in animal models due to the short lifespan of rodents and ethical reasons.

Epidemiological studies have attempted to investigate if cancer incidence or cancer mortality is increased in populations experiencing pain, but the data is discrepant and heterogenous, both in terms of study populations and exposure definitions. For instance, cancer incidence was increased in participants reporting widespread pain in a prospective study, and musculoskeletal pain was associated with higher subsequent cancer rates in older people [4, 5]. In contrast, some studies have found no evidence of an association between pain and cancer outcomes [6, 7]. One difficulty in evaluating a potential effect of pain on the risk of cancer lies in the different types of pain, and how complex it is to dissect out pain *per se* from associated components such as inflammation, which promotes tumour growth and metastasis *via* well-characterised mechanisms [8]. Many chronic pain conditions, such as soft tissue injuries, osteoarthritis, or rheumatic disorders, involve inflammatory pathways.

To our knowledge, there is no systematic review of the literature assessing whether chronic pain increases cancer risk. The present study endeavours to fill this gap and to determine if exposure to chronic, primarily non-inflammatory pain increases the risk of cancer incidence or mortality in humans.

## Methods

The review was conducted following the Preferred Reporting Items for Systematic Reviews and Meta-Analyses (PRISMA) guidelines [9, 10]. The protocol of the review was, registered in the Open Science Framework (registration osf.io/ms437).

### Search strategy

The search strategy was developed using the PICO (population, intervention, comparator, outcome) framework. The population of interest are participants that had a minimum of two years with chronic pain and no prior diagnosis of cancer. Continuing chronic pain is the intervention/exposure. Outcomes are compared with populations with no pain and no prior cancer diagnosis. The primary outcomes of the study are cancer incidence and cancer mortality.

Due to conceptual and terminological overlap between pain as an exposure (of interest) and pain as a symptom of cancer (not of interest), preliminary searches indicated that conventional Boolean strategies retrieved a high proportion of non-relevant studies, predominantly focused on cancer-related pain. Given this limitation, a citation chaining approach was employed to improve identification of studies specifically examining pain as a potential exposure preceding cancer outcomes. This approach allowed more targeted retrieval within a conceptually overlapping literature. A seed study was used to identify forward (citing studies) and backwards (cited studies) articles evaluating the effect of pain on cancer mortality or cancer incidence [4]. The search was conducted in Scopus and Web of Science from database inception up to 11/03/2025, and results were combined for screening. Citation chaining was performed until no new relevant articles were found.

### Study selection and characteristics

All studies selected for citation screening, full-text review and extraction were exported into Covidence, and the selection process was conducted according to the recommendations of the Cochrane Handbook for Systematic Reviews of Intervention. The abstract screening was conducted by one reviewer (CL), with a second reviewer (NH) confirming screening results by randomly checking 30% of the 6087 abstracts. No discrepancies were noted. Two reviewers independently conducted full-text review and extraction (CL, SK). Any discrepancies were resolved with the involvement of a third reviewer (MOP).

Full-text, peered reviewed human studies in English were included if a statistical estimate of cancer incidence, cancer mortality or cancer risk following a diagnosis or reporting of chronic, primarily non-inflammatory pain was provided. This included standardised mortality ratios (SMRs), standardised incidence ratios (SIRs), mortality rate ratios (MRRs), incidence rate ratios (IRRs), odds ratios (ORs), and hazard ratios (HRs). The first chronic pain type included was nociplastic pain, including but not limited to, fibromyalgia, complex regional pain syndrome, chronic primary lower back pain, chronic primary headache, chronic primary visceral pain (irritable bowel syndrome, bladder pain syndrome), and chronic, primarily non-inflammatory musculoskeletal pain. The second pain type included was chronic neuropathic pain without an inflammatory component. Studies were excluded if the population had a prior diagnosis of cancer or pain caused by cancer. The exclusion criteria also applied to studies involving pain caused by psychological factors, nociceptive pain (soft tissue injuries) and pain primarily due to inflammation (rheumatic diseases, giant cell arteritis). Publications in languages other than English, non-human studies, and comments or replies that did not provide new data were excluded as well.

### Data extraction and analysis

The extraction tables included study design, chronic pain type, participant number, pain classification, comparator with no pain, follow up times where appropriate, factors adjusted for, cancer incidence or mortality and any statistical estimates. The extraction tables were reviewed and revised by a third reviewer (SS) and any conflicts were resolved by a fourth reviewer (MOP). Statistical estimates of both cancer-related mortality and cancer incidence were largely heterogenous. Meta-analysis was therefore conducted only for HR of cancer-related mortality, while a narrative synthesis was used for the remaining statistical estimates. We performed random-effects meta-regression to explore sources of between-study heterogeneity in HRs for cancer-related mortality. Specifically, musculoskeletal pain subtype (widespread, regional, joint or back pain) was examined as a potential effect modifier of the association between musculoskeletal pain and cancer-related mortality. When studies reported multiple HRs according to pain subtype, sex (male/female), pain frequency (frequent/infrequent), or pain definitions (ACR1990/WP2019), each HR was treated as a separate effect estimate in the analysis. Statistical heterogeneity was quantified using the I^2^ statistics, and between-study variance (τ^2^). Differences between pain subtypes were assessed using a test for group differences.

### Quality assessment

The Newcastle–Ottawa Scale (NOS) was used to assess the quality of the included cohort studies. Each study was evaluated across its selection of cohort, comparability, and outcome to assess the risk for bias. The NOS scores studies on a scale of 0–9, with higher scores indicating better quality. A study with a score of 7–9 was considered high quality, 4–6 as moderate quality and 0–3 is low quality.

## Results

### Study selection

**Fig. 1:**
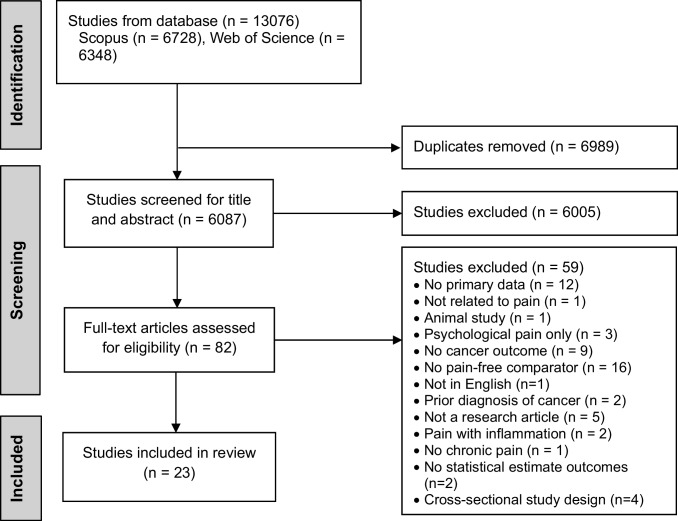
Prisma flowchart of the study selection for inclusion. The flowchart outlines the number of studies through each stage of the review

After completion of the citation chaining, a total of 13,076 studies were identified from two databases. After removal of 6989 duplicates, 6087 studies title and abstract were screened, leading to exclusion of 6005 studies while 82 studies were eligible for full-text screening. A further 58 studies were excluded for reasons shown in Fig. 1. Ultimately, 23 studies were included for the review.

### Study characteristics

The included studies represented a total of 12 prospective cohort studies (98,303 participants, 77,407 comparators) and 12 retrospective cohort studies (451,759 participants, 1,246,542 comparators) (Table 1).

**Table 1:** Summary table of study design

Design	Author, year
Prospective cohort (*n* = 12)	(Andersson, 2009) [11]; (Andorsen, 2016) [12] (Asberg, 2016) [7]; (Ekholm, 2014) [13]; (Holmberg, 2020) [14]; (Macfarlane, 2001) [15]; (McBeth, 2003) [4]; (McBeth, 2008) [16]; (Roseen, 2018) [17]; (Roseen, 2024) [18], (Smith, 2003) [19]; (Lindgren, 2010)[20]
Retrospective cohort (*n* = 11)	(Chen, 2021) [21]; (Dahlqvish, 2024) [22]; (Dreyer, 2007) [23]; (Elliott, 2010) [6]; (Jordan, 2010) [5]; (Jordan, 2013) [24]; (Macfarlane, 2007) [25]; (Macfarlane, 2017) [26]; (Marshall, 2024) [27]; (*Wolfe, 2011) [28]; (Wolfe, 2020)[29]

*Wolfe *et al.* (2011) compared to the general population [28]

### Quality assessment

The Newcastle–Ottawa Quality Assessment (NOQS) identified six out of the 23 cohort studies as high quality [5, 6, 12–14, 21] (Supplementary Table S1). All remaining 17 cohort studies were rated as moderate quality [4, 7, 11, 15–20, 22–29]. Four [12–14, 21] of 10 studies that were included for meta-analysis (40.0%) were of high quality, increasing the validity of the pooled results. Of note, 15 cohort studies had not demonstrated that the outcome of interest was not present at the start of the study, *i.e.* the absence of cancer at baseline was assumed but not clearly identified [7, 11, 12, 16, 17, 20–23, 25–29]. In terms of comparability, only two studies received a nil NOQS score due to the lack of adjustments for potential confounders in its results, where the studies did not adjust for sex or age [23, 24]. Five studies had no statements of follow-up [4, 15, 25–27], which could lead to missing records of cancer mortality or incidence.

### Pain type

A total of nine studies reported on musculoskeletal pain [5, 7, 12, 14, 17, 18, 21, 24, 27]. There were five studies evaluating mixed pain [4, 11, 15, 16, 22]. Three studies each reported on fibromyalgia [23, 28, 29] and chronic pain [6, 13, 19]. Three studies reported on widespread pain [20, 25, 26].

### Overall outcome measures

The review centred on cancer-related incidence and cancer-related mortality. Of the eight studies that reported cancer-related incidence (SIR, IRR, HR, or OR), two studies (25%) showed an increase in cancer incidence (all body sites combined) [4, 5]. Furthermore, five of the total of 23 studies presented data on organ-specific cancers, with only one study showing a statistically significant increase, which was in breast cancer incidence [4]. From a total of 18 studies that reported cancer-related mortality using SMR, MRR, HR, RR or OR, only five (27.8%) studies reported an increased HR, MRR or OR, with chronic pain [4, 15–17, 26]. The majority (*n* = 12, 67%) of the studies indicated that there was no statistically significant effect on the SMR, MRR, HR, RR or OR of cancer-related mortality from chronic pain, with no studies indicating the possibility that chronic pain decreases cancer-related mortality.

#### Cancer-related incidence

There were five types of statistical estimates within the seven studies that presented data on cancer incidence, namely HR, RR, SIR, IRR and OR [4–6, 13, 20, 23, 24] (Supplementary Table S2).

**Fig. 2:**
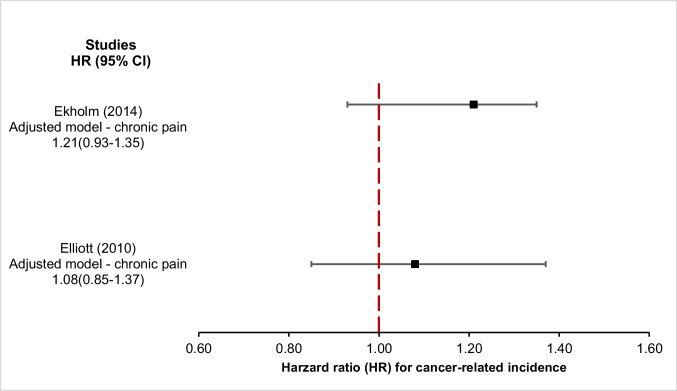
Forest plot of hazard ratio for cancer incidence in relation to observed study effect size

- **Standardised incidence ratio** – Three studies reported SIR [5, 23, 24]; however, only one study included data that accounted for all cancer body sites [23]. This study evaluated cancer incidence among female patients with confirmed and suspected fibromyalgia [23]. Among women with confirmed fibromyalgia, the reported adjusted SIR for overall cancer incidence was 1.2 (95% CI 0.8—1.8) compared to the general population [23]. The other two studies reported SIRs for organ-specific cancers [5, 24].
- **Incidence rate ratio** – One study reported incidence of all malignancies among 8,562 participants with chronic pain compared with 10,073 without chronic pain [4]. The results showed increased cancer incidence in patients experiencing pain (adjusted IRR 1.64, 95% CI 1.23—2.19) [4].
- **Odds ratio** – One study reported OR for cancer incidence in relation to chronic widespread pain and found no significant associations between pain and cancer incidence [20].
- **Hazard ratio** – Three studies reported cancer incidence HR in relation to chronic pain (Fig. 2). In the first study, the adjusted HR for all cancers was 1.08 (99% CI 0.85—1.37), with site-specific adjusted HRs ranging from 0.74 (99% CI 0.40—1.38) for breast cancer to 4.33 (99% CI 0.92—20.47) for lymphoma and leukaemia [6, 13]. In the second one, the adjusted HR for overall cancer incidence among participants with chronic pain compared with those without was 1.21 (95% CI 0.93—1.35) [13]. The third study did not provide the HR for overall cancer but examined pain at specific body sites and reported elevated adjusted HRs for cancer incidence among participants with pain in the back (adjusted HR 1.92, 95% CI 1.17—2.16), shoulder (adjusted HR 1.45, 95% CI 1.19—1.76), and hip (adjusted HR 2.10, 95% CI 1.76—2.50), compared with those without pain [5].

In summary, the evidence on cancer incidence in participants with pain is inconsistent. One study found a slight, non-significant increase in overall cancer incidence in female participants with fibromyalgia, whilst another study found a significantly increased risk in chronic pain participants [4, 23]. HR were generally non-significant, except for one study showing increased cancer incidence in participants with back, shoulder or hip pain [5].

#### Cancer-related mortality

There were five types of statistical estimates within the 18 studies that presented data on cancer-related mortality, namely HR, RR, SMR, MRR, and OR [4, 7, 11–19, 21, 22, 25–29] (Supplementary Table S3).

**Fig. 3:**
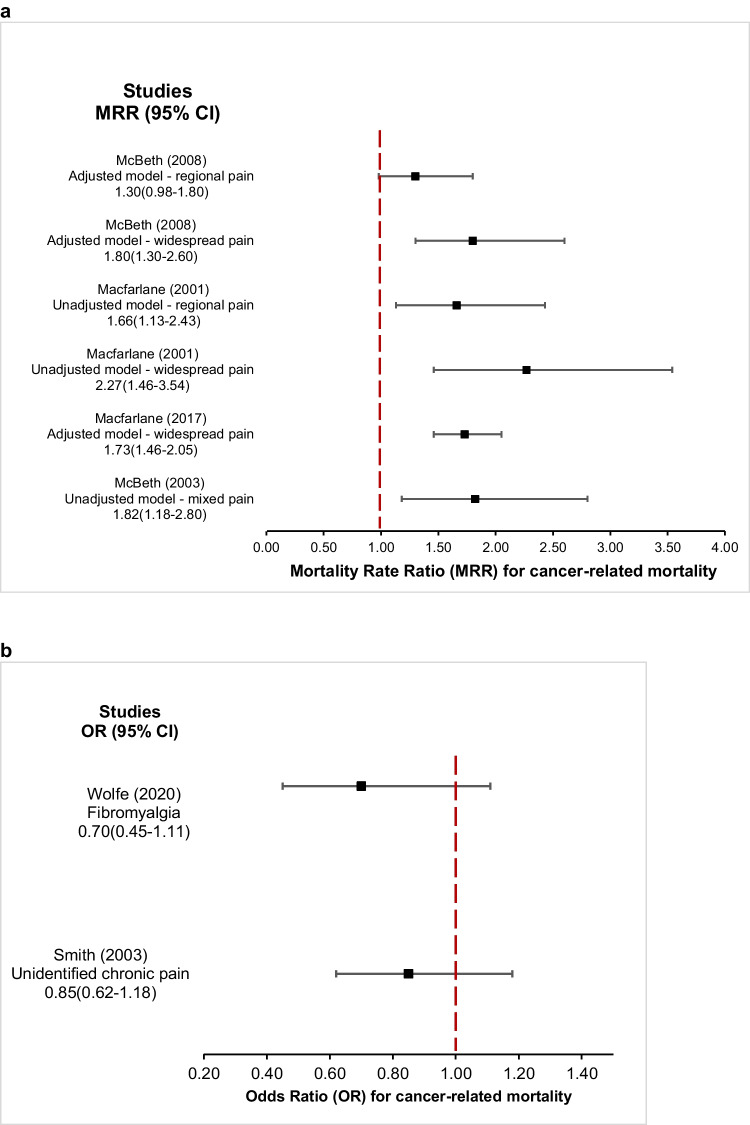
**a**, Forest plot of mortality rate ratio (MRR) for cancer-related mortality, showing observed effect sizes according to pain exposure [4, 15, 16, 26]; **b**, Forest plot of odds ratio for cancer-related mortality, showing observed effect sizes according to pain exposure [19, 29]

**Fig. 4:**
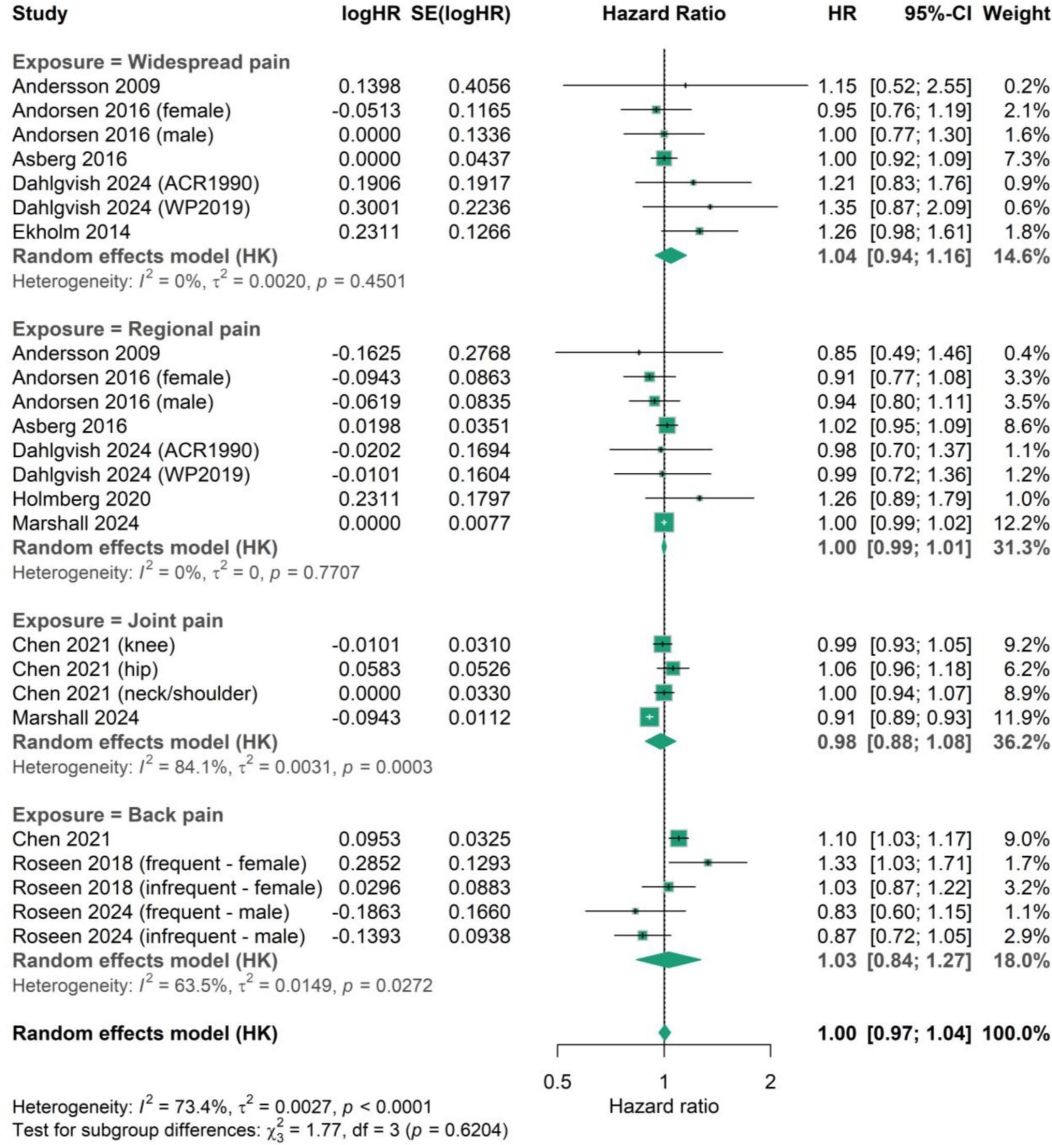
Meta-regression forest plot displaying the association between musculoskeletal pain and cancer-related mortality, with subgroup analyses by musculoskeletal pain types [7, 11–14, 17, 18, 21, 22, 27]

- **Standardised mortality ratio** – Only one study reported cancer SMR, which compared participants with fibromyalgia against the general population [28]. The unadjusted SMR reported for all fibromyalgia patients did not indicate a relationship with cancer mortality (unadjusted SMR 0.95, 95% CI 0.76—1.18) [28].
- **Mortality rate ratio** – A total of four studies described cancer in patients who had either a combination of widespread pain and regional pain (*n* = 2) or widespread pain alone (*n* = 4), resulting in six total MRR data points [4, 15, 16, 25]. The adjusted MRR ranged from 1.30–2.27, with five statistically significant data points despite large variance (Fig. 3a).
- **Odds ratio** – Two studies reported cancer mortality OR [19, 29] (Fig. 3b). The first one (adjusted OR 0.70, 05% CI 0.45—1.11) compared participants with fibromyalgia and the second (adjusted OR 0.85, 95% CI 0.62—1.18) evaluated participants with undefined chronic pain [19, 29]. Both studies suggested a non-significant decrease in cancer-related mortality in patients experiencing pain.
- **Hazard ratio** – All 10 studies [7, 11–14, 17, 18, 21, 22, 27] reporting adjusted HR compared their reported pain-experiencing cohort to a group of participants with no pain, and were included in the meta-regression analysis (Fig. 4). The pooled HR (95% CI) for cancer-related mortality was 1.00 (0.97—1.04) indicating no statistically significant difference between individuals with chronic non-inflammatory pain and those without pain. Substantial between-study heterogeneity was observed in the overall meta-analysis (I^2^ = 73.4%, τ^2^ = 0.0027, p < 0.0001). Meta-regression showed no evidence that the association between musculoskeletal pain and cancer-related mortality differed by pain subtype (test for subgroup differences: χ^2^ = 1.77, p = 0.62). Subgroup analyses demonstrated broadly consistent HRs across pain types, although heterogeneity varied, with pooled HRs (95% CI) of 1.04 (0.94—1.16) for widespread pain (I^2^ = 0%), 1.00 (0.99—1.01) for regional pain (I^2^ = 0%), 0.98 (0.88—1.08) for joint pain (I^2^ = 84.1%), and 1.03 (0.84—1.27) for back pain (I^2^ = 63.5%).
- **Relative risk** – Only one study reported on adjusted relative risk [25]. The study compared 7,182 participants with musculoskeletal complaints to 15,980 participants with no musculoskeletal complaints. It was found that the risk of cancer-related mortality was significantly lower in those with widespread pain compared to those without pain (adjusted RR 0.64, 95% CI 0.46—0.91). Furthermore, participants with regional pain displayed a similar trend of decreased cancer mortality compared to no pain, which was not statistically significant (adjusted RR 0.81, 95% CI 0.64—1.03).

In summary, a discrepant picture emerges from studies reporting mortality: most of them found no significant association between pain and cancer mortality. MRR studies suggested increased mortality in some pain groups. Pooled HRs showed no association between musculoskeletal pain and cancer-related mortality overall [HR (95% CI) = 1.00 (0.97–1.04)] with broadly consistent estimates across pain subtypes. RR data indicated lower cancer mortality in patients with widespread pain (adjusted RR (95% CI) = 0.64 (0.46–0.91)], with regional pain showing a similar, but statistically insignificant trend [25].

### Organ-site specificity

We next investigated whether the effect of pain on cancer risk may be organ-specific, i.e. whether the risk of cancer in some but not all organs is increased. For cancer-related incidence, the following studies reported on organ specific cancers:

- **Breast cancer** – Jordan *et al.* found increased organ-specific cancer-related incidence in patients with pain in different body sites. Female patients with back pain had a higher risk of breast cancer during the first year of follow-up (adjusted SIR 2.12, 95% CI 1.31—3.25) [24]. However, in the same study, the same study population at the tenth year of follow-up showed null increase in breast cancer [24]. Moreover, Dreyer *et al.* reported increased breast cancer incidence in female patients with suspected fibromyalgia (unadjusted SIR 4.8, 95% CI 1.6—11.3), and all female patients with suspected and confirmed fibromyalgia (unadjusted SIR 2.3, 95% CI 1.4—3.7) [23]. Mcbeth *et al.* also reported a significant increase in breast cancer incidence for mixed pain participants (adjusted IRR 3.67, 95% CI 1.39—9.68) [4].
- **Colon cancer** – Colon cancer incidence seemed unaffected from the results of Dreyer *et al.* (unadjusted SIR 1.6, CI 95% 0.2—5.9), Elliott *et al.* (adjusted HR 0.95, 95% CI 0.51—1.77), Jordan *et al.* (adjusted SIR 1.01, 95% CI 0.92—1.10) and McBeth *et al.* (adjusted IRR 2.35, 95% CI 0.96—5.77) [4, 6, 23, 24].
- **Prostate cancer** – Jordan *et al.* revealed that male patients with pain across multiple body sites and follow-up periods showed elevated prostate cancer incidence [24]. During first year follow-up, male patients with new pain diagnoses (adjusted SIR 2.98, 95% CI 2.43—3.62), hip pain (adjusted SIR 5.59, 95% CI 2.56—11.32), neck pain (adjusted SIR 3.53. 95% CI 1.52—6.95), and back pain (adjusted SIR 5.32, 95% CI 3.68—7.43) had a significant increase in prostate cancer incidence [24]. The increased prostate cancer incidence persisted (albeit with smaller effect size) at 10-year follow-up for male patients with new pain diagnoses (adjusted SIR 1.49, 95% CI 1.38—1.60), hip pain (adjusted SIR 1.66, 95% CI 1.13—2.35), neck pain (adjusted SIR 1.80, 95% CI 1.37—2.33), and shoulder pain (adjusted SIR 1.62, 95% CI 1.25—2.09) [24]. However, the other study that reported prostate cancer (adjusted HR 1.26, 95% CI 0.61—2.62) showed a non-statistically significant result [6]. Only McBeth *et al.* reported a significant increase in prostate cancer incidence (adjusted IRR 3.46, 95% CI 1.32—9.59) [4].
- **Lung cancer** – Lung cancer was specified by four studies [(adjusted SIR 2.6, CI 95% 0.0—14.4), (adjusted HR 1.07, 95% CI 0.63—1.81), (Unadjusted SIR 0.99, 95% CI 0.92—1.08),(adjusted IRR 2.04, 95% CI 0.95—4.34)] which did not conclude in an increased incidence [4, 6, 23, 24].
- **Laryngeal cancer**—Laryngeal cancer incidence was increased in all female patients with suspected and confirmed fibromyalgia (unadjusted SIR 1.18, 95% CI 2.1—66.9), and higher in those with confirmed fibromyalgia alone (unadjusted SIR 23.2, 95% CI 2.6—83.7) [23].
- Similarly, **ovarian cancer** [(unadjusted SIR 1.1, 95% CI 0.2—3.1), (adjusted HR 0.90, 95% CI 0.36—2.29)] [6, 23], **bladder cancer** [(unadjusted SIR in all female patients 0.9, 95% CI 0.0—5.1), (adjusted HR 0.83, 95% CI 0.39—1.78)] [6, 23], **oesophageal cancer** (adjusted HR 1.04, 95% CI 0.44—2.46) [6] and **oropharyngeal cancer** (unadjusted SIR 3.2, 95% CI 0.0—19.1) [23], showed no statistically significant increase in incidence with pain.

**Fig. 5:**
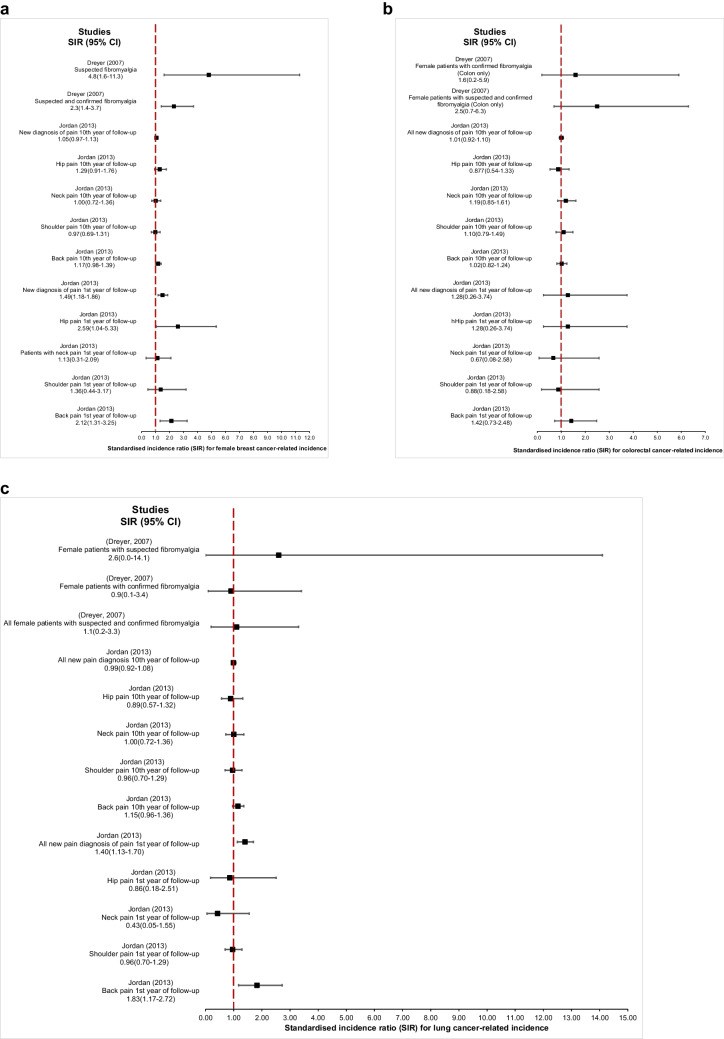
**a**, Forest plot for female breast cancer standardised incidence ratio according to pain types and follow-up times of the studies; **b**, Forest plot for colorectal cancer standardised incidence ratio according to pain types and follow-up times of the studies; **c**, Forest plot for lung cancer standardised incidence ratio according to pain types and follow-up times of the studies

Bladder, prostate, and ovarian cancer results could not be compared in a graphical illustration as the data did not report the same statistical estimates. However, with all studies reporting SIR, lung, breast and colon cancer-related incidence were suitable for forest plot display (Fig. 5). Breast and prostate cancers demonstrated significant associations (Fig. 5a). Female patients with back pain or suspected fibromyalgia had the highest breast cancer incidence (SIR 2.12–4.8) [23, 24], while male patients with new pain or musculoskeletal pain in multiple body sites showed elevated prostate cancer incidence during both short-term and long-term follow-up (SIR 1.49–5.59) [24]. Pancreatic and laryngeal cancers also showed elevated incidence in some studies, whereas other types of cancers generally showed no significant increase.

In terms of cancer-related mortality, two studies reported on lung and one study on prostate, breast and colon cancer [4, 15].

- **Lung cancer** – The only report of organ-specific cancer-related mortality of statistical significance is lung cancer (adjusted MRR 3.09, 95% CI 1.45—6.62) which was reported by Macfarlane et al. [15]. The other study, by McBeth *et al.,* did not support this finding (adjusted MRR 1.66, 95% CI 0.72—3.84) [4].
- **Prostate and large bowel cancer** – McBeth *et al.* reported that both prostate (adjusted MRR 2.81, 95% CI 0.28—27.94) and large bowel (adjusted MRR 1.36, 95% CI 0.42—4.37) cancer mortalities were unaffected in participants experiencing pain [4].
- **Breast cancer** – one study found a non-significant effect on female breast cancer mortality in participants with pain (adjusted MRR 2.45, 95% CI 0.85—7.05) [4].

In summary, body site-specific analyses suggest that chronic pain may be associated with higher incidence for breast and prostate cancers, and with increased mortality for lung cancer.

## Discussion

Our review did not identify a consistent association between chronic pain and overall cancer incidence or mortality, nor did it reveal a clear trend when comparing the data in the light of organ specificity. The meta-analysis of HR for cancer-related mortality showed no significant association with pain, which was largely consistent across widespread, regional, joint and back pain. Nonetheless, the extracted data suggested a slight increase in cancer MRR and incidence of certain site-specific cancers.

Several limitations of the included studies must be considered in interpreting the findings, with reverse causation being a major methodological challenge. Over half of the studies (51.9%) did not explicitly state that the absence of preexisting cancer was verified prior to participant inclusion. This raises the possibility of reverse causation, where chronic pain may have reflected symptoms of undiagnosed cancer, resulting in higher cancer mortality and incidence in the pain group. Reverse causation is also plausible in several studies that reported outcomes over very short follow-up periods. For example, in the study by Jodan et al., the standardized incidence ratio for prostate cancer among patients with different pain types declined from a 3–5 fold increase during the first year of follow-up to a 50%–80% increase after 10 years [24].

Additionally, several cohort studies recruited participants using self-reported pain symptoms. However, this method introduces the risk of bias, where participants may be reporting their pain inaccurately or disproportionally. Lastly, participants experiencing chronic pain are likely to have an impaired ability to exercise, introducing lack of physical activity and increased body weight as possible confounders contributing to increased risk of cancer [4, 23, 30, 31]. These factors may therefore confound the observed relationship between pain and cancer outcomes.

Another limitation of this review is that the selection process may not have adequately accounted for comorbidities, which are particularly prevalent in older populations with chronic pain. In many cases, coexisting conditions may include both inflammatory and non-inflammatory components. This may have led to the inclusion of populations where pain could not be classified as strictly non-inflammatory.

The site-specific increase for high incidence cancers is interesting and may be related to local organ features (*e.g.* immune cell recruitment) or to confounders. The observed increase in breast and prostate cancer should be interpreted with caution. These cancers are commonly detected through screening, and individuals with chronic pain may have greater healthcare utilisation, potentially leading to higher screening rates compared to individuals without pain. As such, the increased incidence observed may reflect increased detection rather than a true elevation in cancer risk.

The inherently tight relationship between physical pain and psychological suffering makes it difficult to isolate the physical pain component in these epidemiological studies. Whilst 91.3% (21/23) of cohort studies adjusted for sex and age, only five studies mentioned the need to consider the psychological state of the participants. Emotional pain and chronic pain often co-exist due to shared cognitive and behavioural mechanisms [32]. Down-regulation of serotonin and norepinephrine, often seen in patients suffering from depression, is linked with pain [33]. Conversely, physical symptoms present in depression include chronic joint pain, back pain and limb pain [33]. This may lead to misclassification of the exposure of the participants. Furthermore, participants with pain experience stress, and chronic stress can elevate inflammatory markers [15, 34]. It is well-known that inflammation increases the risk of cancer, which further consolidates the need to adjust for the psychological state of participants [8, 35].

Analgesic medication use, particularly opioid exposure, represents an important potential confounder in the association between pain and cancer incidence. Individuals with chronic pain are likely to receive opioids, which have been shown to be associated with an increased risk of cancer incidence and mortality in selected organs [36, 37]. Conversely, pain itself is a confounder when studying the effect of opioid on cancer outcomes, reflecting the complex interplay between these factors. Evidence from prospective studies highlights the complexity of this relationship. For instance, a prospective study included in this review compared long-term opioid users, short-term opioid users, non-opioid users with pain and non-opioid users without pain [13]. The findings suggest that non-opioid users with pain had a similar HR to short-term opioid users in terms of cancer incidence and mortality [13, 37]. Conversely, in a recent prospective cohort study participants with chronic pain who reported regular use of opioids had an increased risk of multiple cancer sites, while those with chronic pain who reported use of paracetamol and non-steroid anti-inflammatory drugs (NSAIDs) did not show any increase in cancer risk [36]. These findings highlight the difficulty in studying the independent effects of opioid and pain exposure. The present review did not evaluate opioid use, which represents an important limitation. Future studies aiming to assess the effect of pain on cancer outcomes should account for analgesic exposure to better isolate these interrelated effects.

This review used a non-standard search strategy. Citation chaining was employed as the primary method of study identification. While this approach differs from conventional systematic review methods, it was adopted due to the difficulty in distinguishing between pain as an exposure and pain as a symptom of cancer using standard Boolean search strategies. Citation chaining enabled more targeted identification of relevant studies through reference lists and citation networks, thereby improving the specificity of study selection. However, this approach may have introduced selection bias and limited reproducibility compared to traditional systematic review searches.

Our inclusion criteria referred to “non-primarily inflammatory” pain, to reflect the well documented interplay between chronic inflammation and cancer. Inflammation is considered a hallmark of cancer, with multiple mechanisms converging to initiate or promote cancer (e.g. DNA damage, cellular signalling, immunosuppression and angiogenesis) [35]. Chronic pain is not primarily caused by inflammation in conditions such as fibromyalgia and primarily non-inflammatory musculoskeletal pain [38]. The non-inflammatory component of pain often stems from central sensitisation, where neurons amplify pain signals [38]. However, accumulating evidence shows that central sensitisation is associated with neuroinflammation in the nervous system [39]. This is due to activation of glial cells, and associated release of pro-inflammatory cytokines and chemokines [39]. Fibromyalgia is often a secondary disease after the onset of inflammatory pain conditions, including rheumatoid arthritis, mechanical back pain and visceral pain [38, 40]. Due to the mechanisms of pain pathways in humans, it is difficult to isolate non-inflammatory pain alone without any inflammation component. Therefore, the classification of “primarily non-inflammatory” pain represents an important limitation in this review. In clinical and epidemiological settings, it is difficult to fully exclude the inflammatory components from chronic pain conditions due to overlapping biological mechanisms. Definitions and diagnostic criteria for chronic pain have also evolved over time. ICD-11 introduced a new and pragmatic classification of chronic pain, recognising that oftentimes chronic pain lacks a clearly identifiable aetiology. However, even conditions classified as ‘chronic primary pain’ such as chronic widespread pain and fibromyalgia, may involve inflammatory components. For instance, inflammatory and neuroinflammatory processes may contribute to the pathophysiology of fibromyalgia [41]. Additionally, in chronic musculoskeletal (MSK) pain, while associated with nociceptive processes, an underlying inflammatory component cannot always be excluded. Evidence also suggests that multiple pain mechanisms may co-occur, with individuals often experiencing nociplastic pain alongside neuropathic and nociceptive pain. This overlap highlights the difficulty of classifying chronic pain as strictly ‘non-inflammatory’ [42].

The recent literature on the carcinogenic effect of opium or pharmaceutical opioid consumption [36, 43–46] suggests an increased risk of cancer in certain organs (e.g. lung, pancreas, larynx, bladder) but not others (e.g. breast, colon, kidney, liver, brain). In the current review, mortality and/or incidence of some opium-related cancers, such as lung, pancreatic or laryngeal cancers, were shown to be statistically increased, indicating an interesting overlap between exposure to pain and to opiates in relation to the organs targeted by cancer. However, breast and prostate cancer, whose incidence showed the strongest association with pain, are not in the list of cancers increased in opium or opioid users [36, 43–45]. This partial overlap argues against the notion that the increased risk of cancer in humans taking opioids reflects the pain experienced by the participants. It also points to multiple modes of action for opioid and pain pathways and warrants advanced epidemiology investigations, as well as mechanistic studies into underlying processes.

Several mechanisms have been proposed to underlie the effect of pain on existing tumours, including direct mechanisms (e.g. the effect of pain mediators such as substance P or the innervation of tumours by sensory neurons) and most importantly, indirect mechanisms whereby pain and stress contribute to depressed anti-tumour immunity *via* the hypothalamic–pituitary–adrenal axis and heightened cortisol levels [47–49]. Furthermore, underlying causes for a potential effect of pain on cancer incidence or mortality discussed in the articles we reviewed include hyperresponsiveness to stressors, lack of exercise and being overweight [4, 30, 31]. From the studies included in this review, further mechanisms suggested to explain an effect of pain on cancer include hormonal pathways, psychological conditions, lifestyle habits and comorbidities [4, 11, 15–17, 23, 26].

## Conclusion

This study is the first systematic review of all currently available literature on the association of non-inflammatory pain in cancer-free humans and the risk of cancer mortality or incidence. While the data indicates a small increase in cancer mortality rate ratio, and a possible increased risk of selected organ-site specific cancers (i.e. breast and prostate), it does not support an overall association between pain and the risk of cancer mortality and incidence, and more high-quality studies addressing the limitations of the current literature (e.g. stringent methodologies to address reverse causality and confounding bias) is warranted. This is particularly important because whether pain in humans influences the risk of cancer will determine decision-making in pain management, and especially opioid therapy [36, 45].

## Supplementary Information

Below is the link to the electronic supplementary material.

Supplementary file1 (DOCX 47 KB)

## Data Availability

The datasets analysed in this review are available as supplementary material.
