# Supplementary material for: Risk of cancer incidence and mortality in patients with chronic pain: A systematic review and meta-analysis
Source: Cancer Metastasis Rev. 2026 May 11;45(2):32. doi: 10.1007/s10555-026-10341-2 (PMC13160951; doi:10.1007/s10555-026-10341-2)
Supplement: Supplementary file 1 — Supplementary file1 (DOCX 47 KB) [file 10555_2026_10341_MOESM1_ESM.docx]

**Supplementary Table S1: Quality assessment with the Newcastle-Ottawa Scale (NOS) for cohort studies**

| **Study** | ***Domain 1: Selection*** | | | | **Domain 2: Comparability** | **Domain 3: Outcome** | | | **Total score** | **Quality** |
| --- | --- | --- | --- | --- | --- | --- | --- | --- | --- | --- |
|  | *Representativeness of exposed cohort* | *Selection of non-exposed cohort* | *Ascertainment of exposure* | *Demonstration that outcome of interest was not present at start* |  | *Assessment of outcome* | *Follow-up long enough for outcome to occur* | *Adequacy of follow-up of cohorts* |  |  |
| (Andersson, 2009) | * | * | - | - | * | * | * | * | 6 | moderate |
| (Andorsen, 2016) | * | * | * | - | * | * | * | * | 7 | high |
| (Asberg, 2016) | * | * | - | - | * | * | * | * | 6 | moderate |
| (Chen, 2021) | * | * | * | - | * | * | * | * | 7 | high |
| (Dahlqvist, 2024) | * | * | - | - | * | * | * | * | 6 | moderate |
| (Dreyer, 2007) | - | * | * | - | - | * | * | * | 5 | moderate |
| (Ekholm, 2014) | * | * | * | * | ** | * | * | * | 9 | high |
| (Elliott, 2010) | * | * | - | * | * | * | * | * | 7 | high |
| (Holmberg, 2020) | * | * | * | * | * | * | * | * | 8 | high |
| (Jordan, 2010) | - | * | * | * | * | * | * | * | 7 | high |
| (Jordan, 2013) | - | * | * | * | - | * | * | * | 6 | moderate |
| (Lindgren, 2010) | * | * | - | - | * | * | * | * | 6 | moderate |
| (Macfarlane, 2001) | * | * | - | * | * | * | * | - | 6 | moderate |
| (Macfarlane, 2007) | * | * | * | - | * | * | * | - | 6 | moderate |
| (Macfarlane, 2017) | - | * | - | - | * | * | * | - | 4 | moderate |
| (Marshall, 2024) | - | * | * | - | * | * | * | - | 5 | moderate |
| (McBeth, 2003) | * | * | - | * | * | * | * | - | 6 | moderate |
| (McBeth, 2008) | * | * | - | - | * | * | * | * | 6 | moderate |
| (Roseen, 2018) | - | * | * | - | * | - | * | * | 5 | moderate |
| (Roseen, 2024) | - | * | - | * | * | - | * | * | 5 | moderate |
| (Smith, 2003) | - | * | - | * | * | * | * | * | 6 | moderate |
| (Wolfe, 2011) | - | * | - | - | * | * | * | * | 5 | moderate |
| (Wolfe, 2020) | * | * | - | - | * | * | * | * | 6 | moderate |

**Note:** A study with a score of 7-9 is considered high quality, 4-6 as moderate quality and 0-3 is low quality.

**two marks are rewarded

*a mark is rewarded

-no mark is rewarded

**Supplementary Table S2:** **Extraction table for** **Cancer incidence**

| Author, year | Design of study | Participants | Classification of pain | Comparator | Cancer type | Follow-up time/years of study | N (% of total cancer incidence, WP, RP, NP) | Statistical estimates (OR, HR, SIR, IRR) | Adjusted Factors |
| --- | --- | --- | --- | --- | --- | --- | --- | --- | --- |
| **Odds ratio (OR)** | | | | | | | | | |
| (Lindgren, 2010) | Prospective cohort | 285 (12.5%) subjects with chronic widespread pain (CWP)  588 (24.5%) subjects with chronic regional pain (CRP) | ACR 1990 criteria for fibromyalgia | 1372 (60.2%) subjects with no chronic pain (NCP)  63 (2.8%) subjects cannot be classified | Neoplasms: malignant (ICD10 C00-C97), benign (D00-D89) | 10 years | **Number and percentage of the total 2278 subjects with at least one episode of inpatient care for neoplasms (malignant and benign):** 144 (6.3%) | **OR associated between pain groups and hospitalisation for neoplasm (95% CI):** CWP=1.1(0.6-1.8) p=0.773, CRP=1.1(0.8-1.7) p=0.528, NCP=1 | Adjusted: age, sex, socio-economic, smoking habits and follow-up time |
| **Hazard ratio (HR)** | | | | | | | | | |
| (Elliott, 2010) | Retrospective cohort | Any chronic pain=4135  angina=421, back pain=1279, injury=479  All induvial known to have cancer at baseline were excluded from further analysis. | International Study for the Association of Pain (IASP 1986) | No chronic pain =1718 | Lung, trachea and bronchus (ICD 10 C33 -C34), colorectal (ICD C18-C2), breast (ICD 10 C50), prostate (ICD 10 C61), kidney and bladder (ICD 10 C15-C16), oesophagus and stomach (ICD C15-C16), female reproductive (ICD 10 C53, C54, C56), lymphoma leukaemia (CID 10 C81-C96), other cancers (other 10 ICD codes) | 10 years | **Number of cancer incidence for any chronic pain:** all cancers=451, lung/trachea/bronchus=90, colorectal=64, breast=50, prostate=56, kidney/bladder=37, oesophagus/stomach=34, female reproductive=26, lymphoma/leukaemia=34, other cancers=80  **Number of cancer incidence for no chronic pain:** all cancers=156, lung/trachea/bronchus=32, colorectal=24, breast=27, prostate=16, kidney/bladder=17, oesophagus/stomach=12, female reproductive=11, lymphoma/leukaemia=3, other cancers=21 | **Survival analysis for cancer incidence and chronic pain (Adjusted hazard ratio 99% CI):** all cancers=1.08(0.85-1.37), lung/trachea/bronchus=1.07(0.63-1.81), colorectal=0.95(0.51-1.77), breast=0.74(0.40-1.38), prostate=1.26(0.61-2.62), kidney/bladder=0.83(0.39-1.78), oesophagus/stomach=1.04(0.44-2.46), female reproductive=0.90(0.36-2.29), lymphoma/leukaemia=4.33(0.92-20.47), other cancers=1.41(0.75-2.65)  **Survival analysis for cancer incidence and back pain (Adjusted hazard ratio 99% CI):**  all cancers=1.06(0.78-1.43), lung/trachea/bronchus=1.08(0.55-2.11), colorectal=1.01(0.46-2.21), breast=0.76(0.34-1.69), prostate=1.28(0.52-3.14), kidney/bladder=0.75(0.27-2.11), oesophagus/stomach=1.27(0.44-3.63), female reproductive=0.76(0.22-2.55), lymphoma/leukaemia=3.99(0.72-2.41), other cancers=1.06(0.47-2.41)  angina and injury cause of chronic pain was not collected due to irrelevance | Adjusted: age, sex, except breast, prostate, and female reproductive cancers which were adjusted for age only |
| (Jordan, 2010) | Retrospective cohort | Back=9259, knee=4170, shoulder=3639, neck=3331, hand/wrist=3293, foot=3119, hip=2084, all cases=48206  Patients had consulted with a new musculoskeletal problem in 1996 (no diagnosis in the preceding 2 years) | Morbidity Read Code (or OXMIS code equivalent) within Chapter N plus relevant musculoskeletal codes from Chapters 1, R and S | Controls=40254  Patients with no record of any a musculoskeletal consultation during 1996 and the previous two calendar years | Consultation for malignant or pre-malignant neoplasms under Chapter B of the Read Code hierarchy (Neoplasm); benign neoplasms were excluded | Maximum 10-year follow-up | **Incidence in first year of follow-up, rates per 10000 person-years:** back=280, knee=172, shoulder=195, neck=197, hand/wrist=156, foot=170, hip=252, all cases=222, control=167  **Incidence in 10-year of follow-up, rates per 10000 person-years:** back=2135, knee=2016, shoulder=1977, neck=2032, hand/wrist=1793, foot=1911, hip=2249, all cases=2045, control=1817 | **Effect of comorbidity on risk of mortality during first year of follow-up, adjusted for cancer diagnosis in 2 years prior to baseline or 1 year after baseline HR (95% CI):** back=1.92(1.71-2.16), knee=0.94(0.76-1.17), shoulder=1.45(1.19-1.76), neck=1.13(0.90-1.43), hand/wrist=0.89(0.69-1.15), foot=1.07(0.84-1.37), hip=2.10(1.76-2.50), control=1.00 | Adjusted: diagnosis of cancer in 2 years prior to baseline or 1 year after baseline, age, sex, BMI, drinking, smoking, deprivation status |
| (Ekholm, 2014) | Prospective, | Undefined chronic pain: Non-opioid using participants with chronic pain (CP)=2015 | Respondents with chronic pain were identified through the question  ‘‘Do you have chronic/long-lasting pain lasting 6 months or  more?’’ The question concerning chronic pain was asked in the self-administered questionnaire | Participants with no chronic pain (NP)=10569 | ICD-10 C00–D09 | 6-11 years# (Surveys from 2000 and 2005, 2011 end of follow-up) | **Number of cancer cases:** CP=155, NP=552 | **Hazard ratio (HR 95% CI) of cancer rate according to chronic pain status:** CP=1.15(0.96-1.37), NP=1.00 | Adjusted: age |
| **Standardised Incidence Ratio (SIR)** | | | | | | | | | |
| (Jordan, 2010) | Retrospective cohort | Back=9259, knee=4170, shoulder=3639, neck=3331, hand/wrist=3293, foot=3119, hip=2084, all cases=48206  Patients had consulted with a new musculoskeletal problem in 1996 (no diagnosis in the preceding 2 years) | Morbidity Read Code (or OXMIS code equivalent) within Chapter N plus relevant musculoskeletal codes from Chapters 1, R and S | Controls=40254  Patients with no record of any a musculoskeletal consultation during 1996 and the previous two calendar years | Consultation for malignant or pre-malignant neoplasms under Chapter B of the Read Code hierarchy (Neoplasm); benign neoplasms were excluded | Maximum 10-year follow-up | **Incidence in first year of follow-up, rates per 10000 person-years:** back=280, knee=172, shoulder=195, neck=197, hand/wrist=156, foot=170, hip=252, all cases=222, control=167  **Incidence in 10-year of follow-up, rates per 10000 person-years:** back=2135, knee=2016, shoulder=1977, neck=2032, hand/wrist=1793, foot=1911, hip=2249, all cases=2045, control=1817 | **Incidence in first year of follow-up, SIR (95% CI):** back=1.79(1.57-2.03), knee=1.03(0.80-1.31), shoulder=1.24(0.96-1.57), neck=1.28(0.98-1.64), hand/wrist=0.99(0.73-1.31), foot=1.05(0.78-1.39), hip=1.34(0.99-1.78), all cases=1.36(1.28-1.45), control=1.00  **Incidence in 10-year of follow-up, SIR (95% CI):** back=1.25(1.19-1.32), knee=1.11(1.02-1.20), shoulder=1.13(1.04-1.24), neck=1.20(1.09-1.31), hand/wrist=1.03(0.93-1.13), foot=1.08(0.98-1.19), hip=1.15(1.02-1.29), all cases=1.16(1.13-1.19), control=1.00 | Adjusted: diagnosis of cancer in 2 years prior to baseline or 1 year after baseline, age, sex, BMI, drinking, smoking, deprivation status |
| (Jordan, 2013) | Retrospective cohort | 46656 participants with a recorded musculoskeletal problem in 1996 but not during the previous 2 years (New diagnosis = ND)  AND  8929 participants with back pain (BP)  AND  3525 participants with shoulder pain (SP)  AND  3238 participants with neck pain (NeP)  AND  1998 participants with hip pain (HP) | Diagnosis from a practitioner according to the General Practice Research Database | 39253 subjects with no musculoskeletal consultation in the 2 years prebaseline (NP) | Prostate, breast, lung and colorectal cancer defined as a consultation for a malignant or pre=malignant neoplasm recorded under chapter B of the Read Code hierarchy (“Neoplasms”) or the comparable Oxmis code | Maximum of 10 years | **Number of prostate cancer (men) incidence:** ND=701, BP=142, SP=61, NeP=59, HP=31, NP=414  **Number of breast cancer (women) incidence:** ND=635, BP=134, SP=41, NeP=42, HP=38, NP=500  **Number of lung cancer incidence:** ND=601, BP=130, SP=44, NeP=41, HP=24, NP=520  **Number of colorectal cancer incidence:** ND=514, BP=96, SP=42, NeP=40, HP=21, NP=438 | **SIR (95% CI) for prostate cancer (men) incidence at 1 year follow-up:** ND=2.98(2.43, 3.62), BP=5.32(3.68, 7.43), SP=1.14(0.24, 3.34), NeP=3.53(1.52, 6.95), HP=5.59(2.56, 11.32), NP=1  **SIR (95% CI) for prostate cancer (men) incidence at 10 years follow-up:** ND=1.49(1.38, 1.60), BP=1.60(1.35, 1.89), SP=1.63(1.25, 2.09), NeP=1.80(1.37, 2.33), HP=1.66(1.13, 2.35), NP=1  **SIR (95% CI) for breast cancer (women) incidence at 1 year follow-up:** ND=1.49(1.18, 1.86), BP=2.12(1.31, 3.25), SP=1.36(0.44, 3.17), NeP=1.13(0.31, 2.09), HP=2.59(1.04, 5.33)  NP=1  **SIR (95% CI) for breast cancer (women) incidence at 10 years follow-up:** ND=1.05(0.97, 1.13), BP=1.17(0.98, 1.39), SP=0.97(0.69, 1.31), NeP=1.00(0.72, 1.36), HP=1.29(0.91, 1.76), NP=1  **SIR (95% CI) for lung cancer incidence at 1 year follow-up:** ND=1.40(1.13, 1.70), BP=1.83(1.17, 2.72), SP=1.51(0.65, 2.97), NeP=0.43(0.05, 1.55), HP=0.86(0.18, 2.51), NP=1  **SIR (95% CI) for lung cancer incidence at 10 years follow-up:** ND=0.99(0.92, 1.08), BP=1.15(0.96, 1.36), SP=0.96(0.70, 1.29), NeP=1.00(0.72, 1.36), HP=0.89(0.57, 1.32), NP=1  **SIR (95% CI) for colorectal cancer incidence at 1 year follow-up:** ND=1.06(0.79, 1.40), BP=1.42(0.73, 2.48), SP=0.88(0.18, 2.58), NeP=0.67(0.08, 2.58), HP=1.28(0.26, 3.74), NP=1  **SIR (95% CI) for colorectal cancer incidence at 10 years follow-up:** ND=1.01(0.92, 1.10), BP=1.02(0.82, 1.24), SP=1.10(0.79, 1.49), NeP=1.19(0.85, 1.61), HP=0.877(0.54, 1.33), NP=1 | Adjusted: age, body mass index, smoking status, drinking status, deprivation and comorbidity |
| (Dreyer, 2007) | Retrospective cohort | Female participants with FM=1132  Female participants with possible FM=106  Male participants with FM=57  Male participants with possible FM=25  Total female participants=1269 | ACR 1990 criteria for fibromyalgia | Female participants without FM (including possible cases): 31# | Cancers are classified according to a modified Danish version of the  International Classification of Disease | Mean follow-up of 3.9 years | **Observed numbers of cancer among female participants (all participants) referred to hospital for a diagnosis of FM:** all=43, buccal cavity and pharynx=1, digestive organs=4, colon=4, larynx=2, lung=3, breast=18, reproductive organs=3, urinary organs=1, non-melanoma skin cancer=5, lymphatic and hematopoietic=5, non-Hodgkin’s lymphoma=3, other specified sites=0, secondary and unspecified sites=1  **Observed numbers of cancer among female participants (with confirmed FM) referred to hospital for a diagnosis of FM:** all=27, buccal cavity and pharynx=1, digestive organs=2, colon=2, larynx=2, lung=2, breast=11, reproductive organs=3, urinary organs=1, non-melanoma skin cancer=2, lymphatic and hematopoietic=3, non-Hodgkin’s lymphoma=2, other specified sites=0, secondary and unspecified sites=0  **Observed numbers of cancer among female participants (with possible FM) referred to hospital for a diagnosis of FM:** all=10, buccal cavity and pharynx=0, digestive organs=0, colon=0, larynx=0, lung=1, breast=5, reproductive organs=0, urinary organs=0, non-melanoma skin cancer=2, lymphatic and hematopoietic=2, non-Hodgkin’s lymphoma=1, other specified sites=0, secondary and unspecified sites=0 | **Standardised incidence ratio (SIR 95% CI) of numbers of cancer among female participants (all participants) referred to hospital for a diagnosis of FM:** all=1.5(1.1-2.1), buccal cavity and pharynx=2.7(0.0-15.2), digestive organs=1.1(0.3-2.7), colon=2.5(0.7-6.3), larynx=18.5(2.1-66.9), lung=1.1(0.2-3.3), breast=2.3(1.4-3.7), reproductive organs=0.8(0.2-2.5), urinary organs=0.9(0.0-5.1), non-melanoma skin cancer=1.1(0.4-2.6), lymphatic and hematopoietic=3.9(1.3-9.2), non-Hodgkin’s lymphoma=5.2(1.0-15.1), other specified sites=N/A, secondary and unspecified sites=15(0.0-8.2)  **Standardised incidence ratio (SIR 95% CI) of numbers of cancer among female participants (with confirmed FM) referred to hospital for a diagnosis of FM:** all=1.2(0.8-1.8), buccal cavity and pharynx=3.2(0.0-19.1), digestive organs=0.7(0.1-2.5), colon=1.6(0.2-5.9), larynx=23.2(2.6-83.7), lung=0.9(0.1-3.4), breast=1.8(0.9-3.1), reproductive organs=1.1(0.2-3.1), urinary organs=1.2(0.0-6.7), non-melanoma skin cancer=0.6(0.1-2.0), lymphatic and hematopoietic=3.0(0.6-8.9), non-Hodgkin’s lymphoma=4.4(0.5-15.7), other specified sites=N/A, secondary and unspecified sites=N/A  **Standardised incidence ratio (SIR 95% CI) of numbers of cancer among female participants (with possible FM) referred to hospital for a diagnosis of FM:** all=2.5(1.2-4.6), buccal cavity and pharynx= N/A, digestive organs= N/A, colon= N/A, larynx= N/A, lung=2.6(0.0-14.1), breast=4.8(1.6-11.3), reproductive organs= N/A, urinary organs= N/A, non-melanoma skin cancer=3.2(0.4-11.5), lymphatic and hematopoietic=10.6(1.2-38.2), non-Hodgkin’s lymphoma=12.0(0.2-66.8), other specified sites= N/A, secondary and unspecified sites= N/A | Unadjusted |
| **Incidence Rate Ratio (IRR)** | | | | | | | | | |
| (McBeth, 2003) | Prospective | Mixed pain  (Widespread pain & Regional pain)  6331 with no cancer diagnosis (WP=956, RP=3061) | Widespread pain according to ACR 1990 for fibromyalgia  Regional pain according to ACR 1990 criteria with no fibromyalgia | Participants having no pain (NP=2314) | **N (90+107 with WP):** all malignancies=86, all malignancies excluding nonmelanoma skin cancer=80, female breast=10, prostate=5, lung=19, gastric and small intestine=6, large bowel=12, all other cancers=28  **Site specific analysis – incidence:** all malignancies=395, all malignancies excluding nonmelanoma skin cancer=310, female breast=41, prostate=29, lung=55, gastric and small intestine=16, large bowel=39, all other cancers=130 | 9 years | Prospective | **Location and risk adjusted IRR (95% CI):** WP= 1.61(1.21-2.13), RP=1.19(0.94-1.50), NP=1  **Site specific analysis – IRR (95% CI), NP=reference group:** all malignancies=1.64(1.23-2.19), all malignancies excluding nonmelanoma skin cancer=1.83(1.32-2.54), female breast=3.67(1.39-9.68), prostate=3.46(1.25-9.59), lung=2.04(0.96-4.34), gastric and small intestine=1.59(0.35-7.24), large bowel=2.35(0.96-5.77), all other cancers=1.18(0.70-2.00) | Adjusted: age, sex, study location- (proxy measure of socioeconomic status) |

**Abbreviations:** ACR=American College of Rheumatology, FM=fibromyalgia, WSP=widespread pain, RP=regional pain, HR=hazard ratio, OR=odds ratio, SIR=standardised incidence ratio, IRR=incidence rate ratio, RR=risk ratio, MSC=musculoskeletal complaints, WMSC=widespread musculoskeletal complaints, NP= no pain, KP=knee pain, HP=hip pain, BP=back pain, NSP=neck or shoulder pain, NeP=neck pain, SP=shoulder pain

**Note:**

#calculated values

**Supplementary Table S3:** **Extraction table for** **Cancer-related mortality**

| **Study** | **Cohort design type** | **Chronic pain type and participant number** | **Pain defined by** | **Comparator**  **Patients with no pain and no prior diagnosis of cancer** | **Follow-up time** | **Outcomes** | | **Adjusted factors** |
| --- | --- | --- | --- | --- | --- | --- | --- | --- |
|  |  |  |  |  |  | **N (% of total cancer)** | **Statistical estimates (HR, OR, SMR, MRR)** |  |
| **Hazard ratio (HR)** | | | | | | | | |
| Andersson, 2009 | Prospective | Mixed pain   - Chronic pain (CP)=888 - Widespread pain (WSP)=152 - Regional pain (RP)=736 | Questionnaire | Participants with no chronic pain (NP)=721 | 13.5 years | **Number of deaths per 1000 person years due to malignancy:** WSP=4.26, RP=2.96, NP=2.88 | **Adjusted HR 95% CI:** WSP=1.15(0.52-2.55), RP=0.85(0.49-1.45) | Age, sex |
| Dahlqvish, 2024 | Retrospective | Mixed pain  2425 (M: 1132, F: 1293); mean (SD) age 46.5 (15.4) yrs  ACR1990: CWP=303, CRP=588  WP2019: CWP=200, CRP=686  No mention of whether participants have previous cancer diagnosis  CWP: chronic widespread pain  CRP: chronic regional pain | Questionnaire | Participants with no chronic pain ACR1990 and WP2019: NCP=1466  NCP: No chronic pain | 25 years | Total death irrespective of cancer: 608 of 2425  ACR1990: 577 | **ACR1990**   - **HR(95%CI):** WP=1.21(0.83-1.76), RP=0.98(0.70-1.36), NP=1 - **Adjusted HR(95%) for neoplasm (C00-D48):** WP=1.14(p=0.472), RP=1.02(p=0.913) - **Adjusted HR(95%) for malignant neoplasms of digestive organs (C15-C26):** WP=1.93(p=0.034), RP=1.01(p=0.971)   **WP2019**  **HR(95%CI):** WP=1.35(0.87-2.09), RP=0.99(0.72-1.35), NP=1  **Adjusted HR(95%) for neoplasm (C00-D48):** WP=1.28(p=0.249), RP=0.99(p=0.970)  **Adjusted HR(95%) for malignant neoplasms of digestive organs (C15-C26):** WP=1.88(p=0.094), RP=1.21(p=0.501) | Age, sex, smoking habits, socioeconomic status |
| Ekholm, 2014 | Prospective | Undefined chronic pain   - Chronic pain (CP) in non-opioid users=2015 | Questionnaire | Participants with no chronic pain (NP)=10570 | 6 years# (surveys from 2000 and 2005, 2011 end of follow-up) | **Number of cancer mortality:** CP=85, NP=250 | **Hazard ratio (HR 95% CI) of cancer mortality according to chronic pain status:** CP=1.26(0.98-1.61), NP=1.00 | Age |
| Andorsen, 2016 | Prospective | Musculoskeletal pain   - Musculoskeletal complaint (MSC)=9639 - Widespread musculoskeletal complaint (WSP)=3445 | Questionnaire | Participants with no musculoskeletal complaint (NP)=17309 | 21 years | **Total number of deaths from cancer (%):** MSC=578(6.0), WSP=218(6.3) | **Adjusted model 2 hazard ratio (HR 95% CI) among female participants with MSC or WSP:** MSC=0.91(0.77-1.08), WSP=0.94(0.80-1.11)  **Adjusted model 2 hazard ratio (HR 95% CI) among male participants with MSC or WSP:** MSC=0.94(0.80-1.11), WSP=1.00(0.77-1.30) | Age, smoking, mental health complaints, educational level, body mass index, leisure time physical activity, self-reported chronic diseases  (cancer, CVD, diabetes or asthma) |
| Asberg, 2016 | Prospective | Musculoskeletal pain   - Musculoskeletal complaints (MSC)=31210 - Widespread musculoskeletal complaints (WMSC)=15075 | Questionnaire: CMSC defined according to ACR1990 with some modification (section 2.2) | Patients with no chronic musculoskeletal complaints (No CMSC)=33816 | Mean of 14.1 years | **N of cancer mortality:** No CMSC=1542, CMSC=1936, CMWSC=924 | **Adjusted model 1 HR (95% CI):** No CMSC=1.00, CMSC=1.05(0.99-1.13), CWMSC=1.06(0.97-1.15)  **Adjusted model 2 HR (95% CI):** No CMSC=1.00, CMSC=1.02(0.96-1.09), CWMSC=1.00(0.93-1.09)  **Adjusted model 3 HR (95% CI):** No CMSC=1.00, CMSC=1.03(0.96-1.10), CWMSC=1.02(0.94-1.11)  **Adjusted model 4 HR (95% CI):** No CMSC=1.00, CMSC=1.02(0.95-1.09), CWMSC=1.00(0.91-1.08) | Adjusted model 1: age, sex  Adjusted model 2: sex, education, smoking, self-reported cardiovascular disease  Adjusted model 3: age, sex, education, smoking, self-reported cardiovascular disease, alcohol, body mass index, systolic blood pressure, metabolic syndrome  Adjusted model 4: age, sex, education, smoking, self-reported cardiovascular disease, alcohol, body mass index, systolic blood pressure, metabolic syndrome, physical activity, Hospital Anxiety and Depression Scale |
| Chen, 2021 | Retrospective | Chronic musculoskeletal pain   - knee pain (KP)=37002 - hip pain (HP)=10163 - back pain (BP)=33731 - neck or shoulder pain (NSP)=31331 | Questionnaire : Musculoskeletal pain was defined using the options in the UK Biobank touchscreen questionnaire (Category 100,048) which includes headache, facial pain, neck or shoulder pain, back pain, stomach or abdominal pain, hip  pain, knee pain and pain all over the body | Participants with no pain (NP)=197098 | Mean of 7.4 years | **Number of deaths due to cancer:** KP=1301, HP=388, BP=1172, NSP=1000, NP=6502 | **Adjusted multivariate model (HR 95% CI):** KP=0.99 (0.93-1.05), HP=1.06(0.96-1.18), BP=1.10(1.03-1.17), NSP=1.00(0.94-1.07)  NP=1.00 | Adjusted: age, sex, townsend deprivation index, ethnicity |
| Holmberg, 2020 | Prospective | Participants with musculoskeletal pain within last 14 days (MSK) =1971 | Questionnaire: 97 itemed and 42 were related to musculoskeletal conditions | Participants with no musculoskeletal pain within last 14 days (NP) =2835 | Mean of 19.1 years | **Number of deaths due to cancer:** MSK=176, NP=177 | **Adjusted (1: age and sex) hazard ratio (HR 95% CI) in relation to MSK pain within last 14 days for cancer mortality:** MSK=1.34(1.09-1.65), NP=1  **Adjusted (2: age, sex, marital status, contact to friends or acquaintance, and education level) hazard ratio (HR 95% CI) in relation to MSK pain within last 14 days for cancer mortality:** MSK=1.31(1.06-1.61), NP=1  **Adjusted (3: age, sex, marital status, contact to friends or acquaintance, and education level, physical activity, stress in daily life and comorbidity) hazard ratio (HR 95% CI) in relation to MSK pain within last 14 days for cancer mortality:** MSK=1.26(0.89-1.80), NP=1 | Adjusted 1: age, sex  Adjusted 2: age, sex, marital status, contact to friends or acquaintance, educational level  Adjusted 3: age, sex, marital status, contact to friends or acquaintance, educational level, physical activity, stress in daily life, comorbidity |
| Marshall, 2024 | Retrospective | Musculoskeletal pain  Participants with regional pain (RP)=173621# | Questionnaire: Patients aged more than or equal to 45 years  with a first ever record of one of our index illnesses and with more than or equal to 24 months prior registration at their practice to obtain consultation  records for musculoskeletal pain | Participants with no musculoskeletal pain (NP)=634478#  #calculated | Mean of 3.3 years | **Number of cancer cases by types of musculoskeletal pain:** RP=68339, NP=291256 | **Adjusted associated mortality with type of musculoskeletal pain by cancer model 3 (HR 95% CI):** RP=1.00(0.98-1.01), NP=1.00 | Model 3: age, number of medications, excluding analgesia |
| Roseen, 2018 | Prospective | Chronic Musculoskeletal pain  Women aged 65 or older with three categories of back pain   - Frequent back pain (FBP)=779 - Infrequent persistent back pain (IPBP)=3622 | Questionnaire | Women aged 65 or older  with no back pain (NP)=2001 | Mean of 14.1 | **Death rate per 100000 person-years:** FBP=1128, IPBP=865, NP=798 | **Hazard ratios (HR 95% CI) for association of back pain measure with cancer mortality rate model 3:** FBP=1.33(1.03-1.71), IPBP=1.03(0.87-1.23), NP=1.00 | Model 3: age, education, marital status, living alone, recruitment site, health-related characteristics: excellent general health, current smoker, obsess, prevalent vertebral fracture, arthritis, hip pain, fall history, hospitalisations, hypertension, previous stroke, diabetes, history of breast cancer, previous breast surgery anxiety medication use |
| Roseen, 2024 | Prospective | Chronic Musculoskeletal pain  Male participants with three categories of back pain   - Frequent back pain (FBP)=370 - Persistent back pain (PBP)=2204 | Questionnaire: conducted exam at baseline and year 5 | Male participants with no back pain (NP)=1192 | Mean of 10.3 | **Death rate per 100000 person-years:** FBP=1613, IPBP=1383, NP=1519 | **Hazard ratios (HR 95% CI) for association of primary back pain measure with cancer mortality rate among older men in MrOS cohort model 2:** FBP=0.83(0.60-1.15), IPBP=0.87(0.72-1.04), NP=1.00 | Model 2: sociodemographic measures included in Model 1 and additional health characteristics, including self-reported general health,  obesity, depressive symptoms, smoking status, alcohol status, prevalent vertebral fracture, fall history, arthritis, hip pain, chronic obstructive pulmonary  disease, hypertension, diabetes, cancer history |
| **Odds ratio** | | | | | | | | |
| Smith, 2003 | Prospective | Undefined chronic pain  Female participants with chronic pain=38.4%, 3868#  Total participants=10073 | Survey by participants | Female participants with no pain (NP)=8.9%#, 896# | 27-33 years# | N/A | **Odds ratio (OR 95% CI) between pain and cancer:** CP=0.85(0.62-1.18), NP=1.00 | Adjusted: age, social class, smoking |
| (Wolfe, 2020) | Retrospective | FM  Clinically diagnosed FM (FM)=3659  Participants with FM according to FM 2016 criteria (FM+)=9300 | ACR 1990 for WSP  FM2016 | Participants with no FM according to FM2016 (FM-)=25984 | Mean of 9.3 years | **Total mortality:** 1902 | **Odds ratio (OR 95% CI) according to FM2016:** FM=0.70(0.45-1.11), | Adjusted: age, sex |
| **Standardised mortality rate** | | | | | | | | |
| (Wolfe, 2011) | Retrospective | Fibromyalgia  Participants with FM=8186 | The fibromyalgia ness scale, the  Widespread Pain Index (WPI), widespread pain  (from the ACR 1990 criteria definition), and fibromyalgia  diagnosis modified from the ACR 2010 diagnostic  criteria | General population | Mean of 7.3 years | **Number of fibromyalgia patient deaths due to malignant neoplasms (%):** 22.2, n=1817# | **Standard mortality rate (SMR 95% CI) for all fibromyalgia patients:** 0.95(0.76-1.18) | Unadjusted |
| **Mortality risk rate** | | | | | | | | |
| (McBeth, 2003) | Prospective | Mixed pain  (Widespread pain & Regional pain)  6331 with no cancer diagnosis (WP=956, RP=3061) | Widespread pain according to ACR for fibromyalgia  Regional pain according to ACR with no fibromyalgia | Participants having no pain (NP=2314) | 9 years | **n (90+107 with WP):** all malignancies=86, all malignancies excluding nonmelanoma skin cancer=80, female breast=10, prostate=5, lung=19, gastric and small intestine=6, large bowel=12, all other cancers=28 | **MRR (95% CI), NP=reference group:** All malignancies=1.82(1.18-2.8), all malignancies excluding nonmelanoma skin cancer=1.90(1.21-2.96), female breast=2.45(0.85-7.05), prostate 2.81(0.28-27.94), lung=1.66(0.72-3.84), gastric and small intestine=1.78(0.34-9.29), large bowel=1.36(0.42-4.37), all other cancers=0.85(0.38-1.89) | Adjusted: age, sex, study location- (proxy measure of socioeconomic status) |
| (Macfarlane, 2017) | Retrospective | Widespread pain  500434 (WP=7130) | ACR 1990 for fibromyalgia | Participants having no chronic pain (NP=281718) | 5-9 years# | **n:** cancer=7486(58%) for both WP and NP | **Adjusted MRR (95% CI): of WP vs NP** cancer=1.73(1.46-2.05) | Adjusted: age, sex and excluding first 2 years of follow up in case they had undetected cancer |
| (Macfarlane, 2001) | Prospective | Mixed pain  (Widespread pain & Regional pain)  Total=6569  (WP=1005(15%), RP=3176(48%))  236 participants had been diagnosed as having cancer | ACR 1990 for fibromyalgia | Participants having no pain (NP=2388(36%)) | 8 years | **n in WP sub-cohort free from cancer diagnosis at time of original survey:** WP=42, RP=88 | **MRR (95% CI) from WP in sub-cohort free from cancer diagnosis at time of original survey:** WP=2.27(1.46-3.54), RP=1.66(1.13-2.43)  *NP forms reference group  Site-specific MRR  Upper GI=2.21(0.43-1.31), lower GI=3.25(0.75-14.01), lung=3.09(1.45-6.62) | Adjusted: age, sex, study  Older and more women patients (adjusted) |
| (Mcbeth, 2008) | Prospective | Mixed pain  (Widespread pain & Regional pain)  Patient with widespread pain (WP)=761  Patient with regional pain (RP)=1590 | ACR classification criteria for FM | Patients with no pain (NP)=1993 | 8 years | **Number of deaths due to all cancers:** total=228 | **Adjusted mortality risk ratio (MRR 95% CI):** WP=1.8(1.3-2.6), RP=1.3(0.98-1.8), NP=1.00 | Adjusted: age, sex, Townsend score, practice and ethnic group |
| **Relative Risk** | | | | | | | | |
| (Macfarlane, 2007) | Retrospective | Total of 7182 participants  Participants with widespread body pain (WSP)= 20% females (1436.4) and 12% in males (861.84) | ACR 1990 for fibromyalgia | Participants with no musculoskeletal complaints (NP)=15980 | 14-16 years | **Total number of deaths due to cancer:** 339 | **Risk ratio (RR 95% CI) for death due to cancer in relation to pain:** WSP=0.64(0.46-0.91), RP=0.81(0.64-1.03), NP=1 | Adjusted: age group, gender, education, physical work stress, mental work stress, alcohol consumption, tobacco smoking, BMI |

- **Abbreviations:** ACR=American College of Rheumatology, FM=fibromyalgia, WSP=widespread pain, RP=regional pain, HR=hazard ratio, OR=odds ratio, SMR=standardised mortality ratio, MRR=mortality rate ratio, RR=risk ratio, MSC=musculoskeletal complaints, WMSC=widespread musculoskeletal complaints, NP= no pain, KP=knee pain, HP=hip pain, BP=back pain, NSP=neck or shoulder pain, FBP=frequent back pain, IPBP=infrequent persistent back pain

**Note:** #calculated values
